# Thermal stability of levopimaric acid and its oxidation products

**DOI:** 10.1186/s13065-023-01031-z

**Published:** 2023-09-20

**Authors:** Yuanlin Li, Hongqin Chen, Heng Yan, Yangyong Xu, Jinwen Tang, Runsen Wang, Mengru Yan, Yuqiao Dai, Yongguang Huang, Xiongmin Liu

**Affiliations:** 1 College of Liquor and Food Engineering, Key Laboratory of Fermentation Engineering and Biological Pharmacy of Guizhou Province, Guizhou University, Guiyang, 550025 Guizhou China; 2 College of Chemistry and Chemical Engineering, Guangxi University, Nanning, 530004 Guangxi China; 3 Guizhou Tea Research Institute, Guiyang, 550000 Guizhou China

**Keywords:** Reaction progress, Thermal decomposition, Oxidation characteristics, Thermal oxidation, Peroxide value

## Abstract

Biofuels are renewable alternatives to fossil fuels. Levopimaric acid‒base biofuels have attracted increasing attention. However, their stability remains a critical issue in practice. Thus, there is a strong impetus to evaluate the thermal stability of levopimaric acid. Through thermogravimetry (TG) and a custom-designed mini closed pressure vessel test (MCPVT) operating under isothermal and stepped temperature conditions, we investigated thermal oxidation characteristics of levopimaric acid under oxygen atmosphere. Thin-layer chromatography (TLC) and iodimetry were used to measure the hydrogen peroxides generated by levopimaric acid oxidation. A high pressure differential scanning calorimeter (HPDSC) was used to assess hydroperoxide thermal decomposition characteristics. Gas chromatography-mass spectrometry (GC-MS) was used to characterize the oxidation products. The thermal decomposition kinetics of levopimaric acid were thus elucidated, and a high peroxide value was detected in the levopimaric acid. The decomposition heat (Q_DSC_) and exothermic onset temperature (T_onset_) of hydroperoxides were 338.75 J g^−1^ and 375.37 K, respectively. Finally, levopimaric acid underwent a second-stage oxidation process at its melt point (423.15 K), resulting in complex oxidation products. Thermal oxidation of levopimaric acid could yield potential thermal hazards, indicating that antioxidants must be added during levopimaric acid application to protect against such hazardous effects.

## Introduction

Pine oleoresin is a valuable nontimber biomaterial that is collected from the resin canal of *Pinus* Linn [1–3]. Pine oleoresin is a mix of abietic-type resin acids (~ 75%) and terpene-based neutral compounds (~ 15%), which mainly comprise levopimaric acid (~ 35%). The levopimaric acid molecule comprises conjugated nonsaturating bonds and tri-rings with a carboxylic group [4–6]. Levopimaric acid has recently attracted attention for its uses in biofuels [7]. Levopimaric acid could be directly used as a hydrocarbon fuel [8, 9]. Levopimaric acid could be used as raw material to synthesize epoxidized corn oil with good performance [10]. Under temperature less than 333 K, photo-sensitized oxidation reaction of 2-amino-2-methyl-1-propanol salt of levopimaric acid could generate peroxides [11].

In addition, it could improve the performance of biofuels as a value-added fuel additive [12–14]. Gum rosin is produced at an estimated 1,200,000 tonnes per year worldwide, and its use as a raw industrial material has been widespread. Levopimaric acid, however, presents a tricky challenge to use in applications: the conjugated linkages in the levopimaric acid molecule are unstable. They can be destabilized by contact with oxygen, heat, metal ions, and light, which leads to thermodynamic instability and performance degradation.

Several studies have explored the oxidation characteristics of levopimaric acid. Li et al. found that rosin acid could generate hydroperoxide during oxidation [15]. Under adiabatic conditions, Liu et al. observed oxygen absorption and exothermic phenomena during levopimaric acid oxidation using an accelerating rate calorimeter (ARC) [16]. The molecular structure of levopimaric acid has a homocyclic conjugated double bond, which easily reacts with oxygen and forms high concentration peroxides. A two-step thermal oxidation process for levopimaric acid was described by Ren et al.: (1) formation of peroxides from levopimaric acid and (2) secondary oxidation products resulting from the breaking of the unstable OO bond of peroxides [17].

Nevertheless, very few studies have examined the thermal stability of levopimaric acid peroxide. The mechanism inducing lipid self-oxidation is known as a free radical chain reaction with peroxide as the main product [18]. The peroxide’s weak O–O bond, which contains thermal potential, makes it unstable and easily decomposed [19]. Bulk rosin may be exposed to high temperatures and oxygen during production. For example, a facility producing pine oleoresin caught fire on December 12, 2005, in Guangxi, China, leaving one worker missing and one injured [20]. Careful thermal stability assessment of levopimaric acid oxidation as well as hydroperoxide production is crucial [21].

A key issue is thus to evaluate the thermal stability of levopimaric acid and resolve its potential thermal hazards to enable its promise as a biofuel. We conducted a TG experiment to obtain the nonisothermal thermal decomposition kinetics of levopimaric acid in an oxygen atmosphere. Levopimaric acid oxidation was monitored using a high-sensitivity micro closed pressure vessel testing system (MCPVT) under both stepped and isothermal heating settings. Thin-layer chromatography (TLC) was used to separate the levopimaric acid hydroperoxide that was produced in light of its thermal instability. The high pressure differential scanning calorimeter (HPDSC) test was used to assess the thermal safety parameters of separated levopimaric acid peroxides. Additionally, possible reaction pathways were identified based on the oxidation products. Our research offers insights into the thermal stability of levopimaric acid for the development of rosin-based biofuels.

## Materials and methods

### Materials

Pine oleoresin was purchased from Guangxi Wuzhou Arakawa Chemical Industries, Ltd. Purification of levopimaric acid from rosin base was performed using Qi’s method (97.0 wt%) [22]. Adamas-beta Chemical Company provided chloroform (99.7 wt%), CDCl_3_ (99.8 wt%), silica gel (99.5 wt%), and KI (99.5%). Tetramethylammonium hydroxide pentahydrate (97 wt%) and Na_2_S_2_O_3_ (99.5 wt%) were obtained from Aladdin Industrial Corporation. Nanning Yunlaida Gas Corporation provided the N_2_ and O_2_ (99.999%) gas. Yantai Xinnuo Corporation provided GF 254 silica gel plates. N-Pentane (97.0 wt%), diethyl ether (99.5 wt%) and acetic acid (99.5 wt%) were obtained from Guangzhou Chemical Reagent.

### Kinetic analysis of levopimaric acid oxidation by TG

The thermal decomposition properties of levopimaric acid under a pure oxygen atmosphere elucidates its molecular structure stability. A thermogravimetric (TG) instrument, SDT Q600, was used to study the non-isothermal thermal decomposition kinetics of levopimaric acid and explore whether an oxygen-adsorption and weight-increment stage of levopimaric acid in an oxygen atmosphere.

TG analysis conditions were as follows: heating range: 303–573 K; heating rate: 0.5, 0.67, 0.83 and 1 K min^−1^; sample size: 10 mg; experimental atmosphere: high-purity oxygen atmosphere. The comparative calculations of Kissinger method and Flynn–Wall–Ozawa (FWO) method were adopted for the experiment data under non-isothermal conditions.

1. The Kissinger method

The Kissinger method obtains the Ea corresponding to the fastest thermal decomposition rate (T_p_). The reaction kinetic equation is given as:

1 $$\frac{d\alpha }{dt}=k\left(T\right)\,f\left(\alpha \right)$$

where α is the degree of conversion of the sample at moment t, t is time, k(T) is the reaction rate constants, f(α) is the most probable dimensionless kinetic function.

Based on the Arrhenius equation, k(T) can be ascribed as:

2 $$k\left( T \right) = Aexp\,\left( { - \frac{{E\alpha }}{{RT}}} \right)$$

Based on $\beta =\frac{{d}_{T}}{{d}_{t}}$, Eq. (1), Eq. (2) and $f\left(\alpha \right)={(1-\alpha )}^{n}$, Eq. 3 is the commonly used Kissinger method model equation:

3 $${ln}\left(\frac{\beta }{{{T}_{p}}^{2}}\right)={ln}\left(\frac{RA}{{E}_{a}}\right)-\frac{{E}_{a}}{R}\frac{1}{{T}_{p}}$$

$\text{ln}\left(\frac{{\upbeta }}{{{\text{T}}_{\text{p}}}^{2}}\right)$ was plotted against 1/T_P_, and linear regression was performed to obtain the intercept and slope of the linear regression curve. The activation energy Ea and pre-exponential factor A are then taken into account.

(2) The FWO method

The thermal decomposition reaction of levopimaric acid is a one-step or multistep complex reaction, and the influence of the reaction progress α on the activation energy during the reaction needs to be considered. Use the heating rates β and the temperature T to obtain the slope and intercept by linear fitting. Ea and A were calculated for various conversion rates based on the value of slope and intercept.

G(α) is the integral mechanism function of the sample at different conversion rates and can be expressed as:

4 $$G\left(\alpha \right)=\frac{A}{\beta }{\int }_{0}^{T}{e}^{-\frac{{E}_{{\upalpha }}}{RT}}dT=\frac{A{E}_{{\upalpha }}}{\beta R}{\int }_{\infty }^{u}\frac{{-e}^{-u}}{{u}^{2}}du=\frac{A{E}_{{\upalpha }}}{\beta R}P\left(u\right)$$

where 0 ≤ u ≤ 60, and P(u) can be calculated by:

5 $$P\left(u\right)={\int }_{\infty }^{u}\frac{{-e}^{-u}}{{u}^{2}}du=0.00484{e}^{-1.0516u}$$

The commonly used kinetic equation of the FWO method is:

6 $${log}\beta ={log}\frac{AE}{Rf\left(\alpha \right)}-2.315-0.4567Ea/RT$$

### Thermal stability of levopimaric acid oxidation by MCPVT

**Scheme 1:**
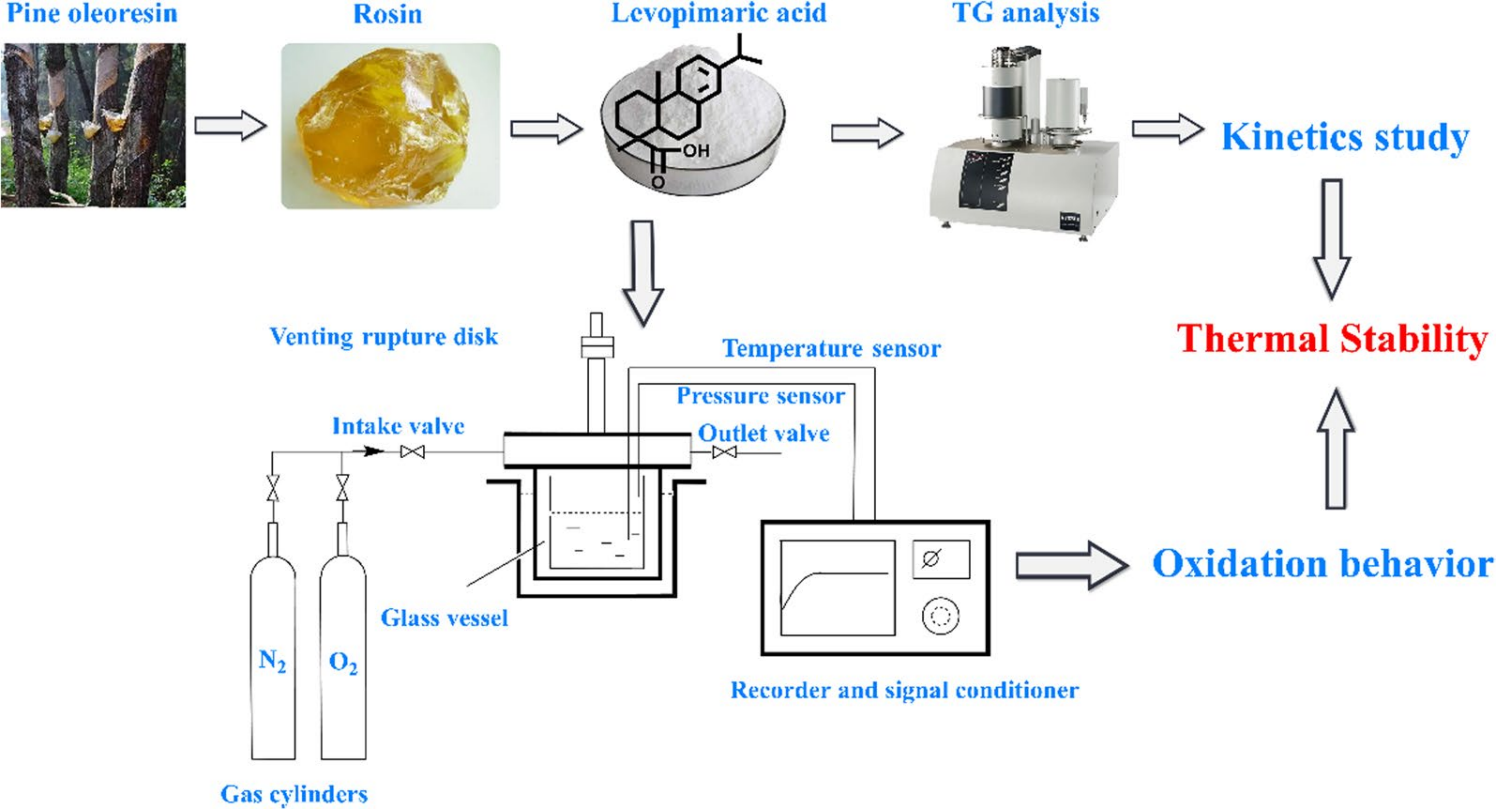
Schematic diagram of the research

Levopimaric acid was tested using a well-controlled MCPVT device. The system comprised three parts, as shown in Scheme 1: a heating furnace, a 35 mL stainless steel container and a temperature/pressure sensor. According to US Recommendations on the Transport of Dangerous Goods, this tool is widely used for monitoring the thermal stability of hazardous chemicals in various conditions [23, 24].

An inner glass liner was set into the container for sample contamination isolation. A glass lining was loaded with 1.18 g levopimaric acid, and the pressure container was sealed. An initial pressure of 0.25 MPa nitrogen/oxygen was propagated from the gas cylinder to the reactor through the inlet valve. During heating, the recorder and signal processor monitored the internal temperature and pressure of the reactor.

The thermal stability of levopimaric acid oxidation process was monitored by the stepwise heating mode of MCPVT: an initial oxygen pressure of 1.2 MPa, 2.4 g of levopimaric acid. Levopimaric acid was oxidized at a constant temperature of 325 K for 23 h, and then step up the temperature. After reaching each set temperature, it would be oxidized at a constant temperature for 2 h. Monitoring and recording the temperature time (T-t) and pressure time (P-t) change curves during the oxidation process. After the reaction is completed, the stainless steel container was quickly cooled to room temperature and collect oxidation products.

### Levopimaric acid peroxide analysis by iodimetry

The oxidation of levopimaric acid was monitored using iodimetry to determine the amount of hydroperoxide produced at different times and temperatures [25, 26]. The reactor was immediately cooled to ambient temperature at the end of the reaction. A solution of potassium iodide (KI) with starch was employed to dissolve the oxidation products. The peroxide produced would be reduced by potassium iodide (Eq. 7) and combined equal quantities of iodine with starch and rendered the solution blue, followed by titrations of the sodium thiosulfate solution until no color was visible (Eq. 8). The results of the hydroperoxide titrations are expressed as milligrams per kilogram (ppm)

7 $$ROOH + \,2KI + \,H_{2} O \to \,2KOH + \,ROH + \,\,I_{2}$$

8 $$2Na_{2} S_{2} O_{3} + \,I_{2} \to 2NaI + \,Na_{2} S_{4} O_{6}$$

### Separation of levopimaric acid peroxide

Peroxide is a highly reactive substance that is the initial oxidation product of various compounds, including lipids, olefins, and ethers. The oxidation products were added to a chromatography column to collect peroxide by using n-pentane and silica gel. Due to the low boiling points of peroxides, we used a 9:1 solution of n-pentane and ethyl ether as the eluent [27]. The eluent was distilled to purify the collected product using a reduced-pressure evaporator at room temperature. Levopimaric acid peroxides were analyzed using thin-layer chromatography (TLC) in this study. Using TLC analysis, a solid‒liquid absorption chromatography method, organic compounds can be separated and quickly identified. Peroxides could be detected by KI-starch solution color variation. Furthermore, TLC analysis took place at ambient temperature, so oxygen‒oxygen bonds were not broken during separation. Using a TLC method, oxidation products were analyzed to clearly identify peroxide species. We used a solution of n-pentane, ethyl ether, and acetic acid (1.5:1:0.05, V/V) as the developing solvent and KI-starch as the chromogenic agent. The product may be considered peroxide as soon as the KI-starch solution produces the corresponding blue stain on the TLC plate.

### Thermal decomposition properties of levopimaric acid peroxide

By using a high-pressure differential scanning calorimeter (HPDSC), we measured the thermal decomposition parameters of the levopimaric acid peroxides. Tests using the HPDSC apparatus are effective in assessing the thermal stability of energetic materials. An instrument equipped with a Q2000 TA DSC was used to conduct dynamic temperature-programmed screenings. A stainless-steel crucible withstood a maximum pressure of 15.0 MPa. Test crucibles were filled with 0.80 mg of levopimaric acid peroxide each and manually sealed. During the dynamic scanning test, a specific 4 K·min^-1^ heating rate was applied to the sample in an atmosphere of nitrogen between 303 and 503 K.

### Analysis of oxidation products

The major intermediate product of the oxidation is peroxide. When in contact with pressure, light or heat, it decomposes into numerous secondary oxidation products. The formed products were evaluated using a GC/MS method [15].

A GC/MS-QP2010 (SHIMADZU, Japan) loaded with an Rxi-5Sil fused silica capillary column (30 m × 0.25 mm × 0.25 μm), and coupled with an electron impact (EI) ionization detector (70 eV), after methyl esterification. The analytical procedures were as follows: heating temperature maintained at 353 K for 3 min, followed by raised to 483 K at a rate of 16 K/min, then with an increase of 5 K/min to 503 K, finally raised from 503 to 513 K with a rate of 4 K/min and was kept for 15 min; the injection temperature and were volume 553 K and 1.0 µL; the split ratio was 70:1; the interface temperature and the ion source temperature were set at 523 K and 503 K, respectively; the quadrupole mass filter was set from m/z 40 to 500 in full scan mode.

## Results and discussion

### Thermal decomposition kinetics of the levopimaric acid oxidation process in an oxidizing atmosphere

**Fig. 1:**
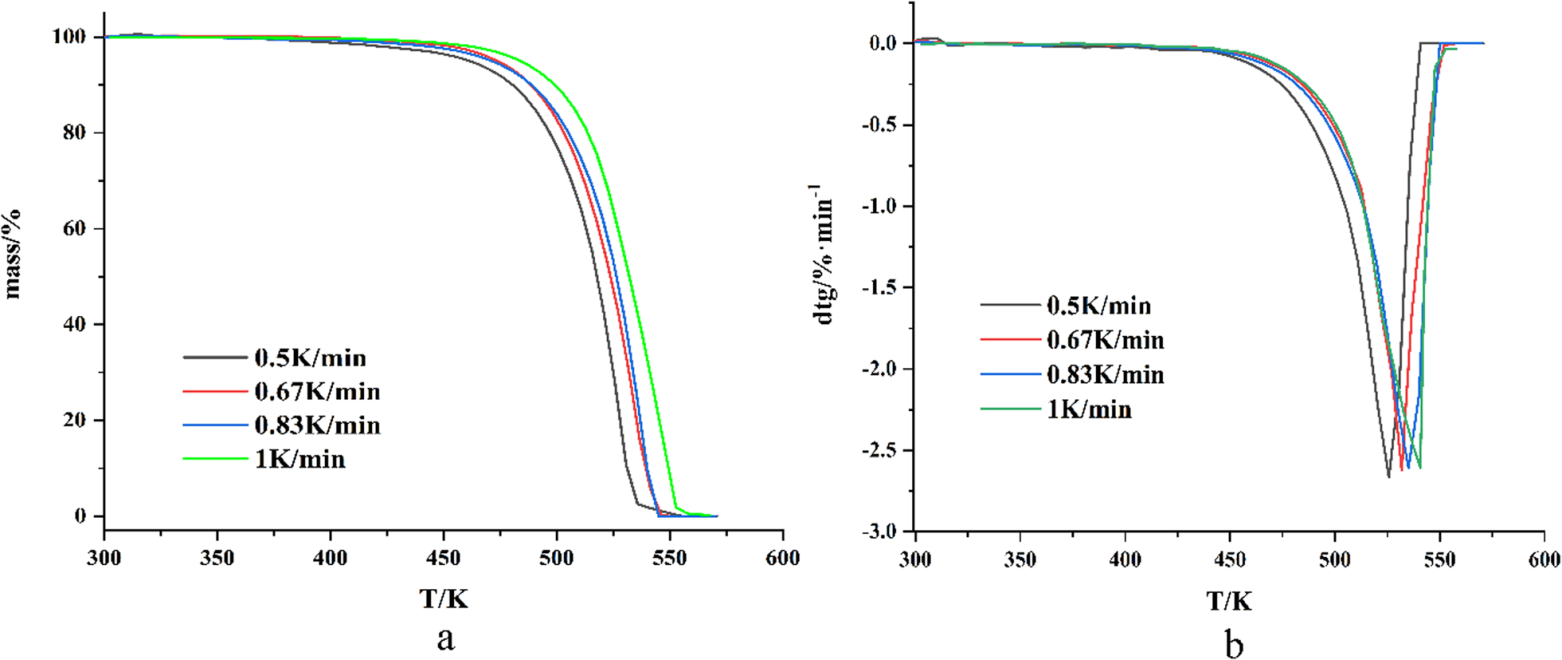
**a** The TG curves of levopimaric acid in an oxygen atmosphere **b** The DTG curves of levopimaric acid in an oxygen atmosphere

The thermal decomposition reaction of levopimaric acid in an oxygen atmosphere was investigated at 303 to 573 K by TG. The TG curves and differential thermogravimetric (DTG) curves are shown in Fig. 1a, b. In Fig. 1a, the thermal decomposition process of levopimaric acid in an oxygen atmosphere only showed a major weight-loss step without an obvious oxygen-adsorption and weight-increment stage. After heating at 530 K, the weight loss of levopimaric acid was almost complete. Levopimaric acid showed similar weight-loss rates at different heating rates, as indicated by the TG curves.

In Fig. 1b, the weight-gain peak at the initial temperature of 303 K on the DTG curve again indicated that levopimaric reacted with oxygen at 303 K. The weight loss of levopimaric acid was a clear step, and the peak temperature was related to the weight-loss step on the TG curve.

In the experimental temperature range, levopimaric acid is not volatile. The thermal decomposition kinetics of levopimaric acid were calculated using two isothermal kinetic models, the Kissinger method and the FWO (Flynn-Wall-Ozawa) method [28]. Regarding these two models, the Kissinger method is a differential method and the FWO method is an integral method, including apparent activation energy, pre-exponential factor, and correlation coefficient [29, 30].

**Fig. 2:**
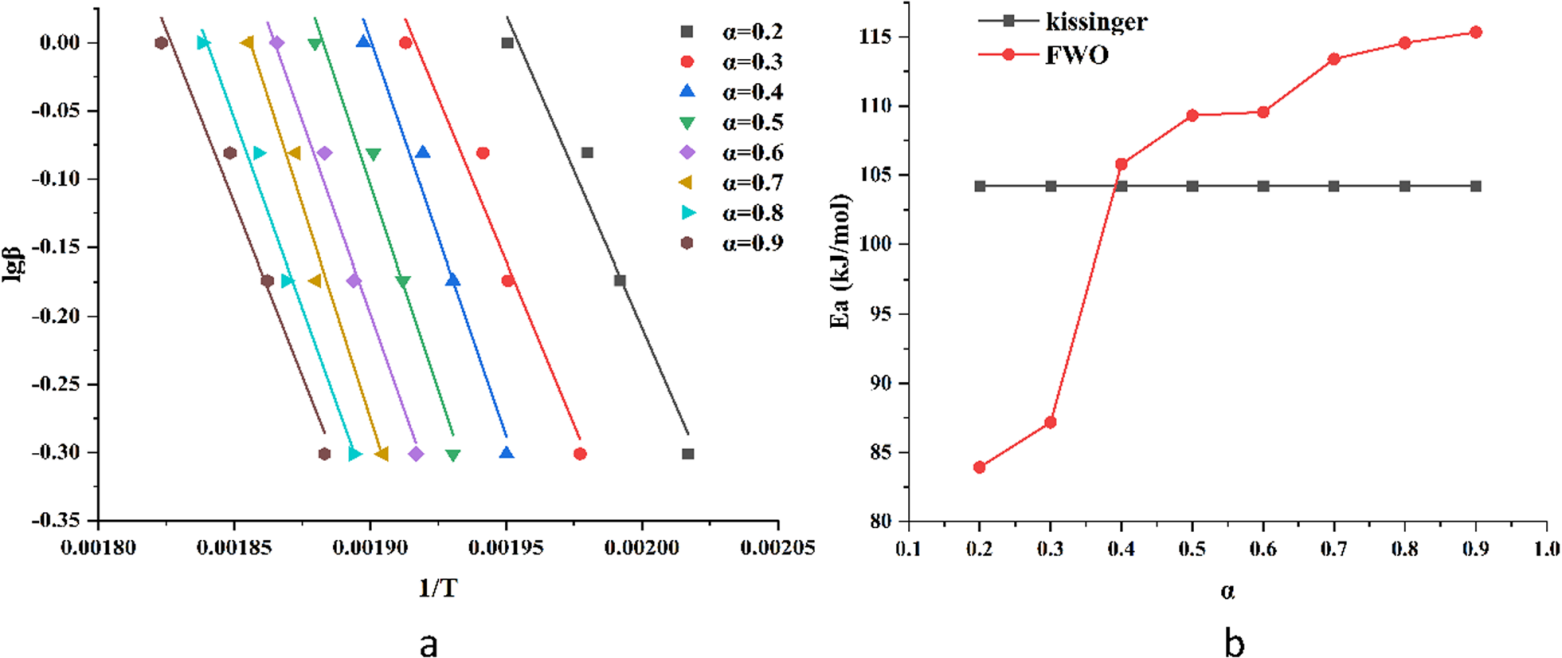
**a** Linear fitting plots obtained by the FWO method of thermal decomposition **b** Ea of levopimaric acid vs. reaction progress with two models

The thermal decomposition activation energy of levopimaric acid obtained by the two kinetic models is shown in Fig. 2 below:

The thermal decomposition activation energy Ea of levopimaric acid obtained by the Kissinger method is 104.20 kJ·mol^−1^ (Table 1). The Ea obtained by the FWO method increased with the progress of the reaction (0.1 to 0.9), wherein the overall trend increased. The widely-used Kissinger method and the FWO method can quickly calculate the Ea value without considering the reaction mechanism to avoid the error that may be caused by assuming different reaction mechanism functions [31]. Compared to other biomass, levopimaric acid shows a relatively lower Ea, which indicates that it has favorable potential as biomass energy [32]. There is only one step on the TG curve, and the mass loss is close to 100%, indicating that the decomposition of levopimaric acid is a violent, complete, and continuous process [33].

**Table 1:** Kinetic parameters at different methods

Method	α	Ea (kJ·mol^−1^)	A (s^−1^)	R^2^
Kissinger	–	104.20	5.16*10^8^	0.987
FWO	0.2	83.93	7.82*10^7^	0.993
	0.3	87.18	1.02*10^8^	0.990
	0.4	105.79	7.96*10^8^	0.984
	0.5	109.31	1.07*10^9^	0.991
	0.6	109.56	9.98*10^8^	0.990
	0.7	113.40	1.43*10^9^	0.987
	0.8	114.56	3.67*10^8^	0.984
	0.9	115.34	1.43*10^8^	0.991

### Pressure behavior of the levopimaric acid oxidation process under isothermal conditions

MCPVT studied the thermal oxidation behavior of levopimaric acid in an oxygen atmosphere by performing isothermal oxidation experiments, and the temperatures and pressures within the reactor were monitored throughout the course of the reaction. Since the MCPVT was a completely closed environment, Eq. 9 is applicable:

9 $$Levopimaric{\text{ }}acid + \,O_{2} \to \,reaction\,products$$

Equation 9 represents a complex oxidation process. According to the result of TG test and the high melting point (423.15 K), levopimaric acid seems to hardly ever causes pressure variations below 413 K. However, monitoring pressure changes allows us to monitor oxygen consumption.

**Fig. 3:**
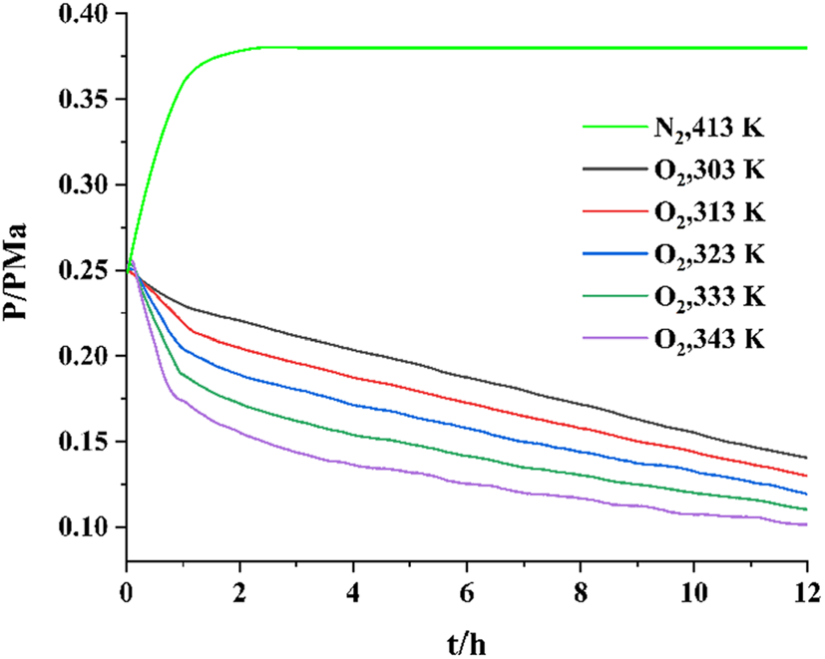
Pressure vs. time of levopimaric acid reaction

During thermal oxidation, levopimaric acid was oxidized in an O_2_ atmosphere. In parallel, a contrast experiment was carried out in a N_2_ atmosphere. While the glass lining was heated to 303 K, 313 K, 323 K, 333 K, and 343 K, the inner temperature and pressure of the reactor were traced. Figure 3 shows these experimental results for pressure versus time (P-t) in a N_2_ atmosphere after heating to 413 K. In pure nitrogen atmospheres, P-t curve is horizontal at 413 K with no pressure change occurred, which means that neither chemical reactions nor phase changes took place below 413 K.

In pure oxygen, the P-t curve atmospheres are not horizontal with pressure drop processes. When levopimaric acid was heated at 303 K, 313 K, 323 K, 333 and 343 K in MCPVT device, it reacted with O_2_, and the oxidation rate increased with the temperature increased. These results were presented by pressure decreased. According to these results, the oxidation process was temperature-dependent and consumed a large amount of O_2_. Table 2 shows the O_2_ consumption of levopimaric acid oxidation based on the number of moles (n) at the beginning (0 h) and end of the reaction (12 h). P and T are calculated as shown in Fig. 3 using the ideal gas equation (n = PV/RT), where R = 8.314 J·mol·K^-1^.O_2_ consumption increased quickly with increasing temperature. At 303 K, levopimaric acid oxidation was investigated with relatively low oxygen absorption, and oxygen consumption was 0.433 × 10^−3^ moles. Preliminary observations suggest that the formation of peroxides between levopimaric acid and oxygen may cause pressure drop under pure oxygen atmosphere, peroxides formation should be carefully checked.

**Table 2:** Oxygen consumption of levopimaric acid at different temperatures

Reaction temperature/K	Oxygen consumption/10^−3^ mol
303	0.433
313	0.461
323	0.484
333	0.506
343	0.522

A pressure drop is shown in Fig. 3 as the oxidation temperatures approach 303 K. At 343 K, the pressure curves showed an even larger drop. Resulting in an oxygen consumption of approximately 0.522 × 10^−3^ mol. Other easily oxidized substances, such as ethers, can be used to study this phenomenon under closed conditions [34]. An explanation could be found in the mechanism of free radical chain reactions [35]. Free radicals are generated during the thermal decomposition of peroxide, which accelerates oxidation to produce complex secondary oxidation products [19]. The formation-decomposition process of peroxides leads to the accumulation of radical pools at a faster rate, resulting in deep radical oxidation of levopimaric acid [36].

### The generation and decomposition of levopimaric acid peroxides

**Fig. 4:**
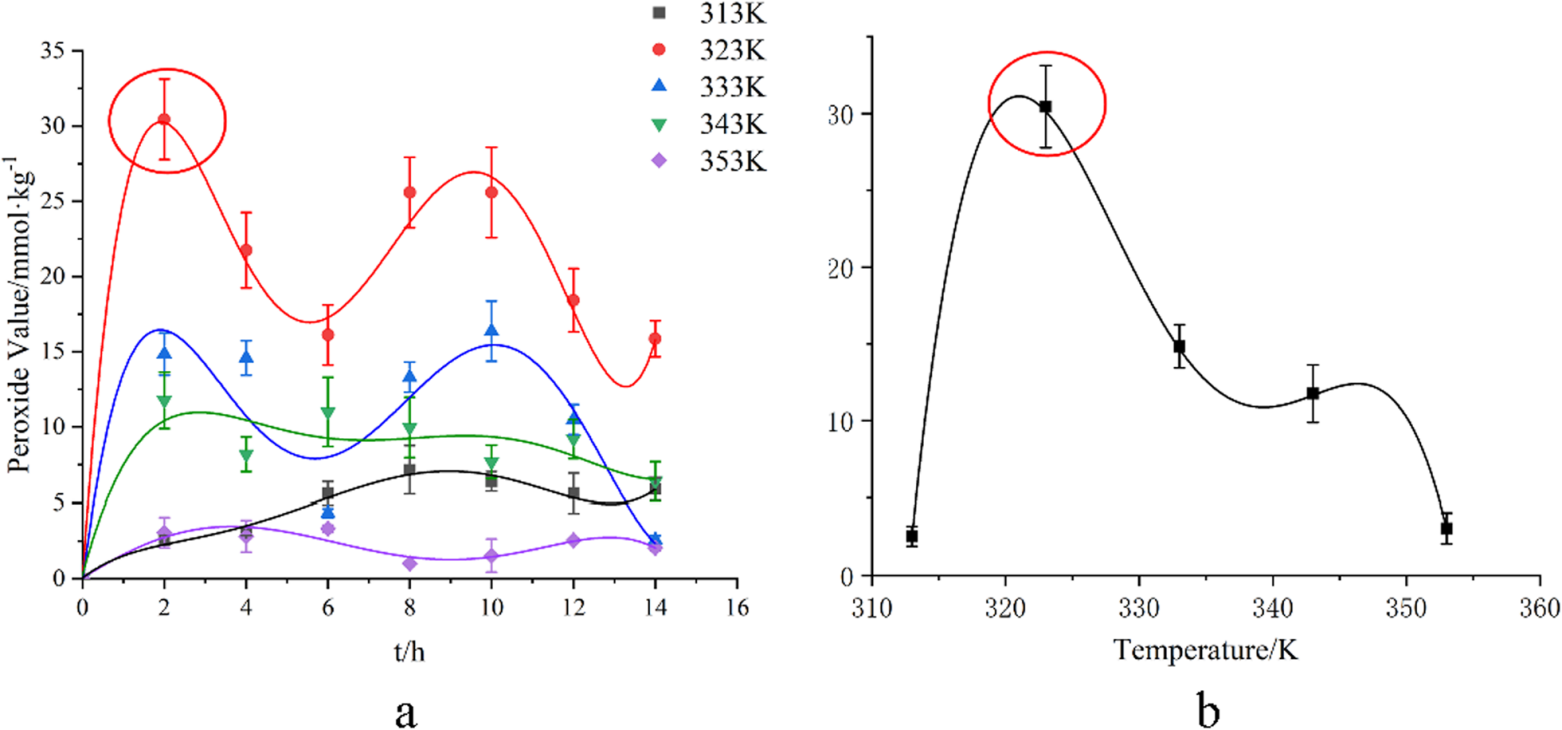
**a** Peroxide value vs. reaction time; **b** Peroxide value vs. reaction temperature at 2 h

Oxygen consumption led to the oxidation of levopimaric acid, followed by peroxide formation and decomposition in the MCPVT results. The generation-degradation process of levopimaric acid peroxides must be further investigated to confirm the results of the reaction, and iodimetry was thus used to determine levopimaric acid hydroperoxide concentrations (peroxide values). Figure 4 shows that the peroxide value changes as a function of reaction time at different temperatures.

When levopimaric acid was oxidized at 323 and 333 K, a high concentration of hydroperoxide formed. Hydroperoxide concentrations increased with time, reaching a maximum of 30.44 mmol·kg^-1^ at 2 h. A large amount of peroxide is formed when levopimaric acid is oxidized [37]. Figure 4 shows the peroxide value-temperature relationship at 2 h. As the temperature increased from 313 to 323 K, the peroxide value increased, indicating the accumulation of peroxides [38]. As the temperature was increased from 323 to 353 K, the value of peroxide decreased significantly. The results showed that the peroxide decomposition rate was faster than that of peroxide because of the instability of O–O bonds [39]. Many free radicals were released to activate levopimaric acid oxidation at depth with oxygen [40]. The iodimetry tests generally fit the MCPVT results fairly well. A large amount of peroxides could be generated during levopimaric acid oxidation. The higher temperature is, the faster peroxides decompose.

### Separation of levopimaric acid peroxide

**Fig. 5:**
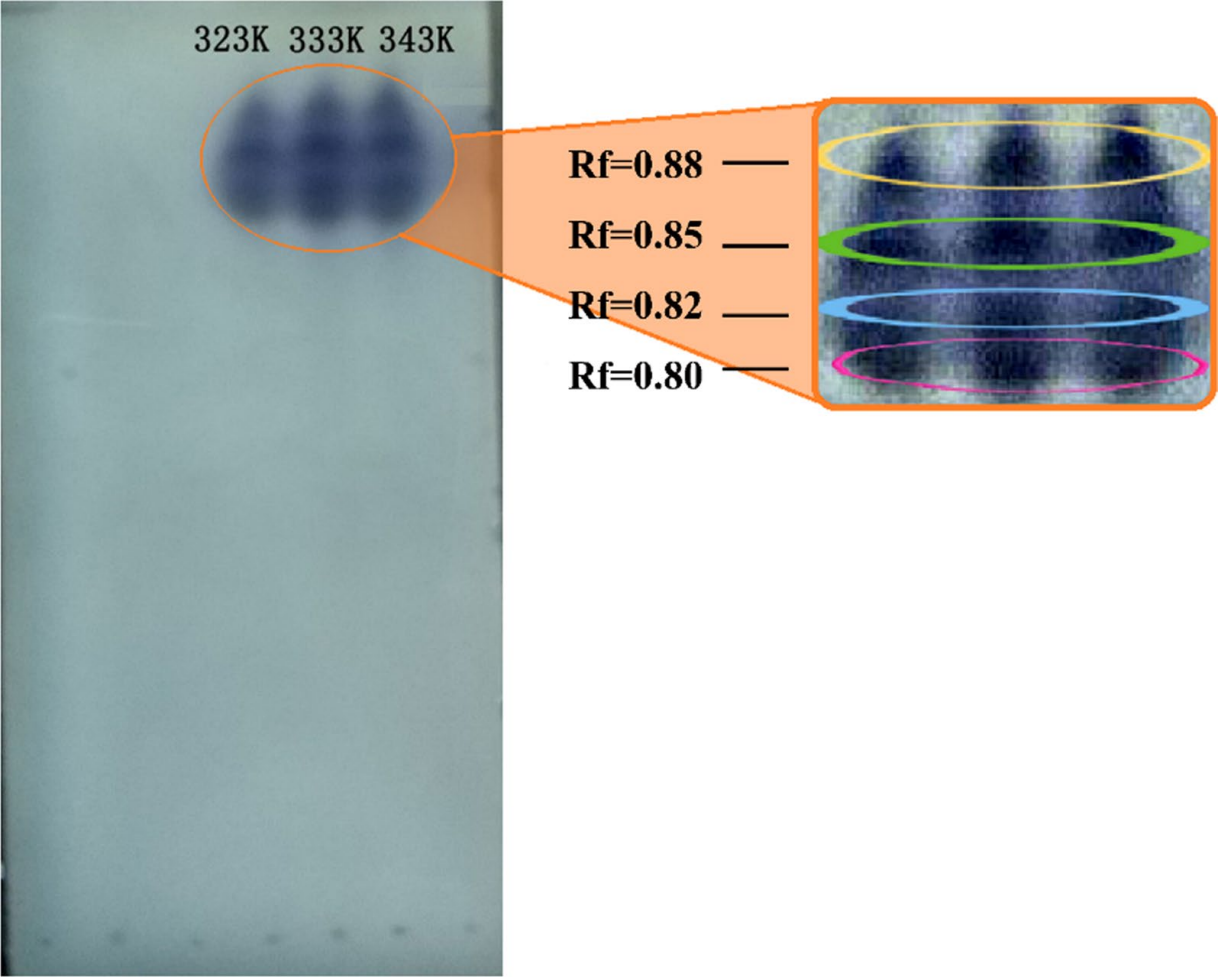
TLC analysis of separated levopimaric acid peroxides

Levopimaric acid relatively easily forms peroxides at high concentrations, a major primary product. Following the commonly accepted mechanism of hydrocarbon oxidation at low temperatures, hydrocarbon oxidation reacts primarily with the ⋅OH radical to yield the R⋅ radical after a short period of initiation, followed by the formation of ROO⋅ radicals with a barrier-free O_2_ addition reaction; ROO⋅ radicals react predominantly only with ⋅HO2 radicals or with the abstraction of H from another molecule to yield ROOH at temperatures ranging from 300 K to approximately 550 K [41]. Much effort has been spent separating levopimaric acid peroxides from oxidation products using column chromatography. TLC analysis was conducted to demonstrate the peroxide species at 323 K, 333 K, and 343 K. After the KI starch solution was dropped, there were four closely spaced blue spots on the TLC plate, as shown in Fig. 5. Four types of peroxide with very similar polarities were generated. Due to their near constant ratio (R_f_) flux values, they are extremely difficult to separate using column chromatography [42]. Ren et al. investigated the oxidation characteristics of levopimaric acid on the basis of two-dimensional infrared spectroscopy and HPLC analysis and found that levopimaric acid reacted with oxygen through a continuous reaction and a parallel reaction starting at 303 K and ending at 323 K [43]. Oxidation products are formed in parallel at low temperature, which may cause the formation of multiple similar polar peroxides of levopimaric acid. It has further demonstrated that levopimaric acid oxidation firstly proceeded through the initial auto-oxidation generating peroxide species, and followed the deep oxidation induced by peroxides decomposition.

### Thermal decomposition of Levopimaric acid peroxides

**Fig. 6:**
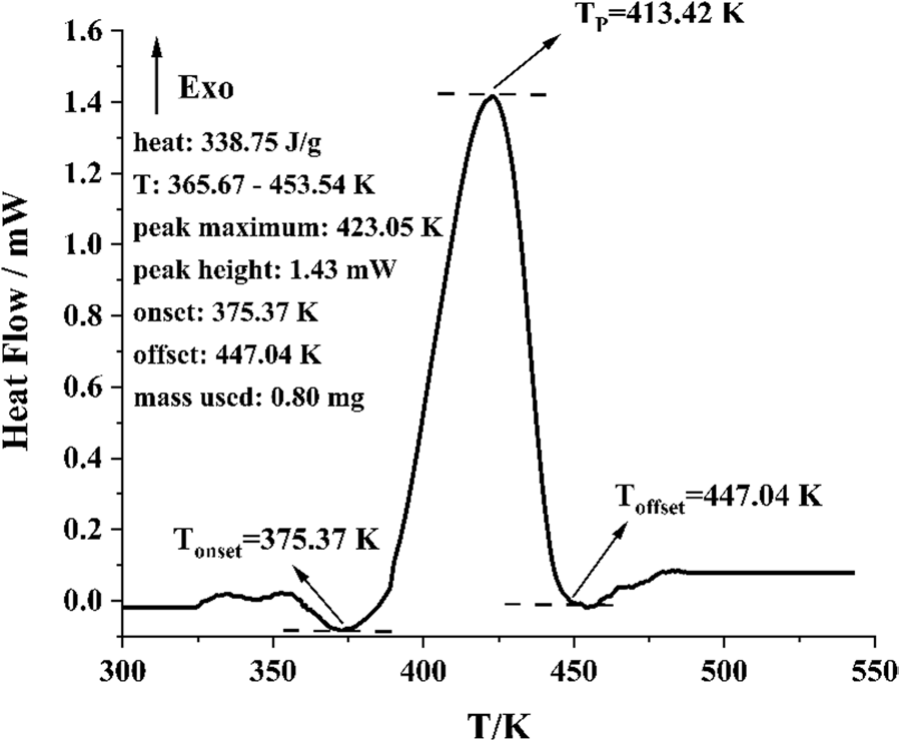
Heat flow vs. temperature of Levopimaric acid peroxides by HPDSC

Therefore, the rudimentary curve of thermal stability and thermal decay is investigated explored, and the HPDSC assay for the Levopimaric acid peroxides that was conducted in the temperature range 303 to 503 K is shown in Fig. 6. Examination of the heat flux curve revealed the presence of a principal exothermic peak from 365.67 to 453.54 K. Its exothermic onset temperature (T_onset_) is 375.37 K and close to the T_onset_ of benzoyl peroxide (377.15 K) [44]. Its thermal decomposition value (Q_DSC_) was 338.75 J·g^-1^ (β = 4 K·min^-1^). This peroxide exhibited a Q_DSC_ of more than 250.00 J·g^-1^ and is expected to be classified as a Category 5 hazard [24]. The HPDSC results in this section were the thermal decomposition of mixtures containing various Levopimaric acid peroxides, which were closer to the actual production of peroxides during storage [45]. Levopimaric acid peroxide was produced extensively during oxidation, which introduced potential hazards to the production and storage processes [46]. In addition to causing potential heat damage, peroxide decomposition releases free radicals that form a wide range of byproducts [47, 48]. Thermal parameters for Levopimaric acid peroxides are shown in Table 3.

**Table 3:** Thermal parameters of Levopimaric acid peroxides by HPDSC at 4 K·min^-1^

Sample mass (mg)	0.80
T_P_ (K)	423.05
T_onset_ (K)	375.37
T_offset_ (K)	447.04
Q_DSC_ (J·g-1)	338.75

### Oxidation behavior of Levopimaric acid under step temperature

**Fig. 7:**
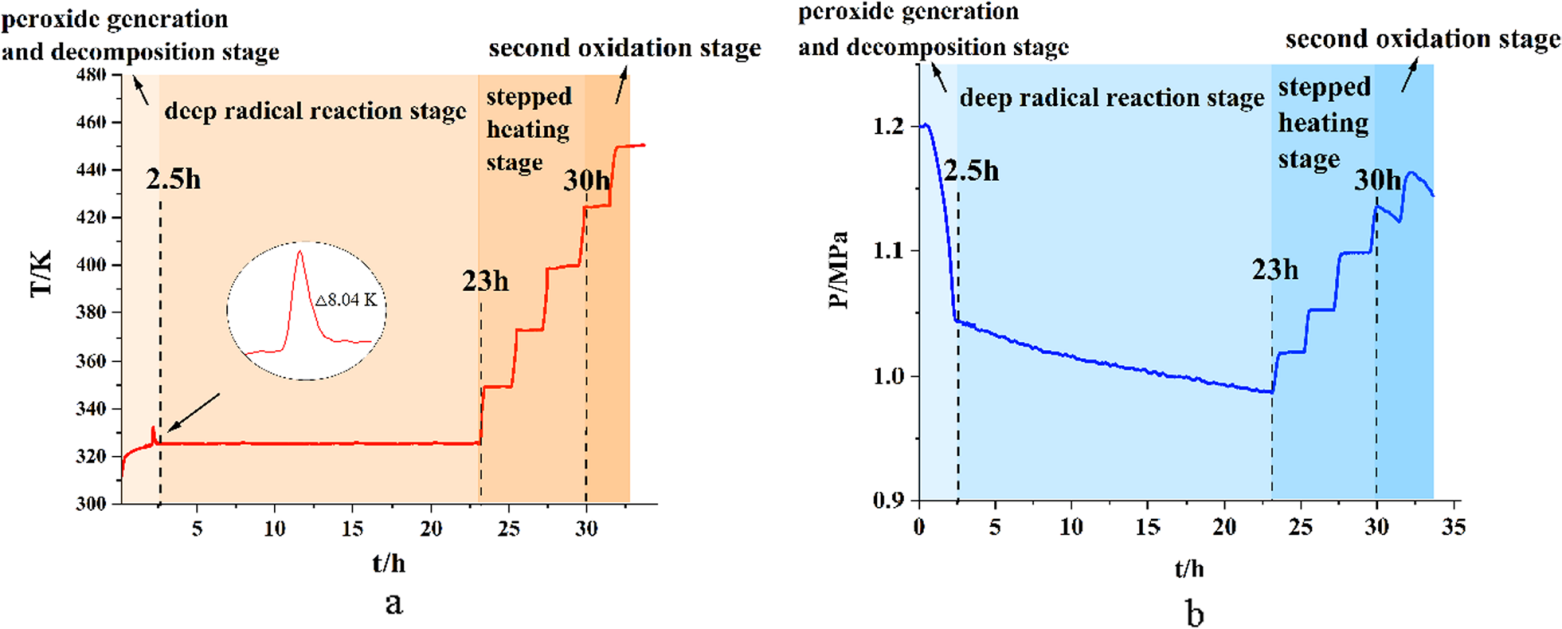
Levopimaric acid stepped oxidation **a** Temperature vs. time; **b** Pressure vs. time

To simulate actual reservoir conditions, a stepwise oxidation test was conducted. Plots of T_t_ (a) and P_t_ (b) from the levopimaric acid oxidation process at the stated temperatures are shown in Fig. 7. There was a decreasing trend in the p-t curve from 0 to 2.5 h, and we observed that levopimaric acid reacted with O_2_ to give rise to a drop in pressure. The T-t curve showed a clear exothermic reaction at 325 K with a sharp temperature peak △T = 8.04 K, and the pressure dropped rapidly. In view of the constant external temperature, the violent exothermic reaction in the system causes the sharp temperature peak [49]. The oxidation process in MCPVT simulates the process of actual storage, which limits dissipation of the decomposition heat of Levopimaric acid peroxide [45].

In the 2.5–23 h time interval, the pressure was lowered by a profound radical reaction between oxygen and levopimaric acid. Radicals were generated in the reaction process via the decomposition of peroxide upon heating. They can easily absorb O_2_ to form various secondary oxidation products [50]. When the oxidation time is approximately 23–30 h, the two curves are almost step lines with no mutation point. Cabaret et al. analyzed the heating process of rosin under different conditions and demonstrated that the oxidized surface outside the rosin acted like amber to prevent further inner rosin reactions [51]. In Fig. 7a, while the temperature reached the melting point of levopimaric acid (423.15 K), an O_2_ consumption ‘reboot’ took place in Fig. 7b. Levopimaric acid’s viscosity value dropped such that oxygen was consumed as the oxidizing surface dissolved in the rosin liquid [52]. As a result, the second stage of oxidation occurred [53].

We used a Risk Matrix method to assess the thermal risk of a chemical process at a defined reaction condition [54]. Table 4 provides a four level hazard rating method using adiabatic temperature rise (ΔT_ad_), and Table 5 uses the Time-to-Maximum Rate under adiabatic conditions (TMR_ad_) to evaluate the possibility of reaction occurrence. A risk matrix for thermal runaway was presented to guidelines for designing risk matrices in Table 6 by multiplying the factors in Tables 4 and 5. When the calculated value is in red zone, the risk could be nonacceptable. When the calculated value is in red zone, the risk could be specified as nonacceptable. When the calculated value is in green zone, the risk could be specified as transitional. When the calculated value is in white zone, the risk could be specified as negligible.

**Table 4:** Assessment Criteria for ΔT_ad_

Severity	ΔT_ad_ (K)	Factor
Negligible	< 50 and no pressure	1
Medium	50 − 200	2
Critical	200 − 400	3
Catastrophic	> 400	4

**Table 5:** Assessment Criteria for TMR_ad_

Probability	TMR_ad (_h)	Factor
Impossible	> 100	1
Remote	50–100	2
Seldom	24–50	3
Occasional	8–24	4
Probable	1–8	5
Frequent	< 1	6

**Table 6.**
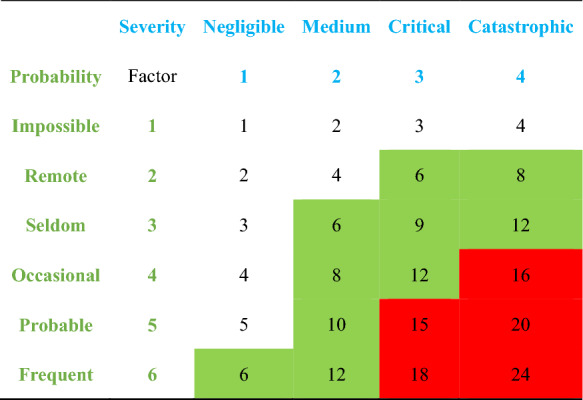
Risk matrix for evaluating reaction thermal runaway

Under the defined reaction condition (2.4 g Levopimaric acid, 1.2 MPa initial O_2_, and 310–325 K temperature), ΔT_ad_ value is 8.64 K, which means the ΔT_ad_ factor is 1; TMR_ad_ value is 2.16 h, which means the TMR_ad_ factor is 5. Herein, the thermal risk is almost negligible due to its value is 5 (white zone).

### Products and possible reaction pathway of levopimaric acid oxidation

**Scheme 2:**
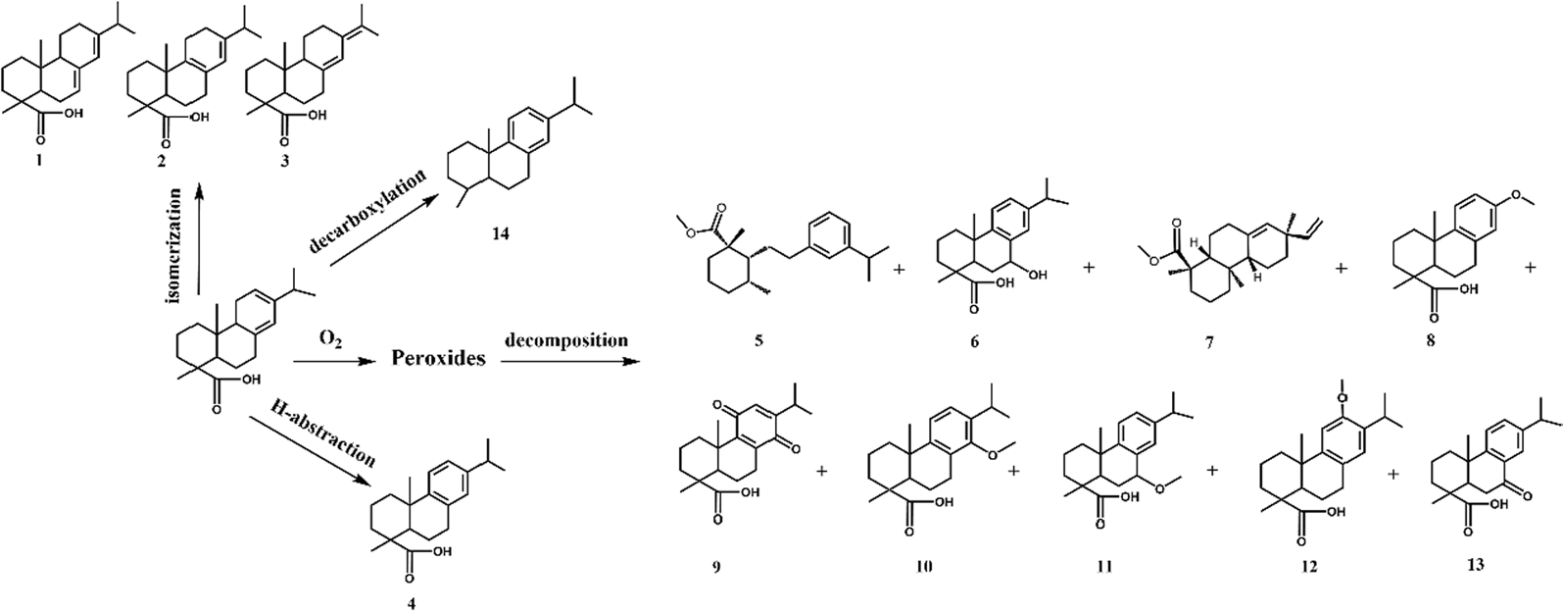
Proposed reaction scheme for levopimaric acid oxidation

In Scheme 2, the number below the molecular structure was corresponding to the rank in Table 7. Table 3 shows that the oxidation products mostly included abietic acid, dehydroabietic acid, 7-Oxodehydroabietic acid, 7-vinyl-1,2,3,4,4a,4b,5,6,7,9,10,10a-dodecahydro1,4a,7-trimethyl-7,15-pimaridiene- methyl 18-phenanthrene 1-carboxylate, palustric acid, neoabietic acid and 1-Phenanthrenecarboxylic acid, 1,2,3,4,4a,9,10,10a-octahydro-6-methoxy-1,4a-dimethyl-7-(1-methylethyl)-, (1R,4aS,10aR)- (ACI). The complete (100%) conversion of levopimaric acid resulted in a large amount of dehydrogenation products and oxygen fixation products.

**Table 7:** Oxidation products of levopimaric acid

NO.	Products	CAS number	Relative content (%)	Similarity (%)
1	Abietic acid	514-10-3	20.78	98
2	Palustric acid	1945-53-5	2.70	95
3	Neoabietic acid	471-77-2	1.95	84
4	Dehydroabietic acid	1740-19-8	51.09	93
5	1,3-Dimethyl-2-[2-[3-(1-methylethyl)phenyl]ethyl]-methylcyclohexanecarboxylate acid	41298-29-7	1.11	86
6	1-Phenanthrenecarboxylic acid, 1,2,3,4,4a,9,10,10a-octahydro-9-hydroxy-1,4a-dimethyl-7-(1-methylethyl)-, (1R,4aS,9R,10aR)- (9CI, ACI)	76235-98-8	1.27	90
7	7-vinyl-1,2,3,4,4a,4b,5,6,7,9,10,10a-dodecahydro1,4a,7-trimethyl-7,15-pimaridiene- methyl 18-phenanthrene 1-carboxylate	1686-54-0	6.95	92
8	13-Methoxypodocarpa-8,11,13-trien-19-oic acid	38041-43-9	1.14	85
9	Methyl 1-Phenanthrenecarboxylic acid, 1,2,3,4,4a,5,8,9,10,10a-decahydro-1,4a-dimethyl-7-(1-methylethyl)-5,8-dioxo-, (1 S,4aS,10aR)- (9CI, ACI)	96160-44-0	1.55	83
10	1-Phenanthrenecarboxylic acid, 1,2,3,4,4a,9,10,10a-octahydro-6-methoxy-1,4a-dimethyl-7-(1-methylethyl)-, (1R,4aS,10aR)- (ACI)	42400-90-8	2.89	85
11	1-Phenanthrenecarboxylic acid, 1,2,3,4,4a,10a-hexahydro-9-methoxy-1,4a-dimethyl-7-(1-methylethyl)-, methyl ester, (1R,4aS,10aR)- (ACI)	197444-13-6	0.98	83
12	1-Phenanthrenecarboxylic acid, 1,2,3,4,4a,9,10,10a-octahydro-6-methoxy-1,4a-dimethyl-7-(1-methylethyl)-, (1R,4aS,10aR)- (ACI)	42400-90-8	0.39	76
13	7-Oxodehydroabietic acid	110936-78-2	4.12	90
14	18-Norabietatriene	19407-17-1	0.03	93
15	Unknown component		1.02	
16	Unknown component		0.77	
17	Unknown component		2.89	

The primary reaction of levopimaric acid is an alkylation reaction with the hydroxyl group radical (·OH), followed by a barrierless reaction with an oxygen molecule to generate the alkyl peroxide radical (ROO·). During the double bond oxidation process from 298 to 550 K, the prevalent pathway of ROO· is to react with ·HO2 radicals or to extract hydrogen peroxide (ROOH), producing H from the organic molecule [55]. As a result, levopimaric acid peroxides are the major product of the initial oxidation. During this oxidation, their thermal decomposition forms many reactive radicals as a rate-determining step [56]. At temperatures above 443.29 K, the peroxides were fully decomposed and became the major source of ·OH, and the radicals would accelerate the accumulation of complicated oxidation products.

The secondary oxidation products of levopimaric acid were 1,3-Dimethyl-2-[2-[3-(1-methylethyl)phenyl]ethyl]-methylcyclohexanecarboxylate acid, 1-Phenanthrenecarboxylic acid, 1,2,3,4,4a,9,10,10a-octahydro-9-hydroxy-1,4a-dimethyl-7-(1-methylethyl)-, (1R,4aS,9R,10aR)- (9CI, ACI), 7-vinyl-1,2,3,4,4a,4b,5,6,7,9,10,10a-dodecahydro1,4a,7-trimethyl-7,15-pimaridiene- methyl 18-phenanthrene 1-carboxylate, 13-Methoxypodocarpa-8,11,13-trien-19-oic acid, methyl 1-Phenanthrenecarboxylic acid, 1,2,3,4,4a,5,8,9,10,10a-decahydro-1,4a-dimethyl-7-(1-methylethyl)-5,8-dioxo-, (1 S,4aS,10aR)- (9CI, ACI), 1-Phenanthrenecarboxylic acid, 1,2,3,4,4a,9,10,10a-octahydro-6-methoxy-1,4a-dimethyl-7-(1-methylethyl)-, (1R,4aS,10aR)- (ACI), 1-Phenanthrenecarboxylic acid, 1,2,3,4,4a,10a-hexahydro-9-methoxy-1,4a-dimethyl-7-(1-methylethyl)-, methyl ester, (1R,4aS,10aR)- (ACI), 1-Phenanthrenecarboxylic acid, 1,2,3,4,4a,9,10,10a-octahydro-6-methoxy-1,4a-dimethyl-7-(1-methylethyl)-, (1R,4aS,10aR)- (ACI), and 7-Oxodehydroabietic acid. Dehydroabietic acid (51.09%) was also detected in large quantities by H abstraction during oxidation. Notably, the yield of abietic acid was found to be relatively high (20.78%), indicating that levopimaric acid is subject to thermal isomerization at temperatures below 450 K. Furthermore, levopimaric acid has also been shown to undergo thermal decarboxylation. We determined that the major product of decarboxylation was 18-Norabietatriene (0.03%) [57].

A simplified scheme for the oxidation of levopimaric acid is proposed in Scheme 2 and combined with the conclusions of previous research [15]. Despite its highly useful properties, biofuel suffers from lower oxidation stability than fossil fuels. Following oxidation stability testing, Viswanathan et al. reported the need to add antioxidants to biofuel [58]. The instability of levopimaric acid molecules with conjugated C = C bonds deserves a great deal of attention. The addition of antioxidants and an inert atmosphere is inevitable for stability [59].

## Conclusions

To investigate its oxidation characteristics and thermal stability in pure oxygen atmospheres, a custom-designed MCPVT was used to monitor the levopimaric acid oxidation process. Based on our results, the following conclusions are made:

1. The thermal decomposition of levopimaric acid is a violent, complete and continuous process. In an oxygen atmosphere, the process of levopimaric acid oxidation occurred over three steps: (1) the initial uptake of oxygen and the generation of peroxides; (2) the thermal decomposition of Levopimaric acid peroxides; and (3) the oxidation of free radicals.
2. The levopimaric acid oxidation reaction resulted in the formation of a high concentration of peroxides. At temperatures below 323 K, the incipient oxidation process proceeded slowly through ·OH-induced H-abstraction, generating the main primary oxidation product: hydroperoxides. These hydroperoxides demonstrated a noteworthy exothermic onset temperature (T_onset_) and decomposition heat (Q_DSC_) of 375.37 K and 338.75 J·g^-1^, respectively.
3. A second-stage oxidation process was demonstrated to occur in levopimaric acid at 423.15 K (the melting point of levopimaric acid) due to the dissolution of the oxidized film. A risk matrix method was used to evaluate the thermal risk of Levopimaric acid oxidation at 2.4 g Levopimaric acid, 1.2 MPa initial O_2_, and 310–325 K temperature. The analysis of this different safety criteria shows that Levopimaric acid oxidation process can be considered safe from a thermal risk viewpoint under the defined reaction condition.
4. In the levopimaric acid oxidation process, complex oxidation products were produced. The antioxidant should be used with levopimaric acid on the basis of this oxidation stability test.

## Data Availability

The datasets utilized and analyzed during this investigation are available upon reasonable request from the corresponding author.
